# Mutation of the TGN1412 anti‐CD28 monoclonal antibody lower hinge confers specific FcγRIIb binding and retention of super‐agonist activity

**DOI:** 10.1111/imcb.12646

**Published:** 2023-04-28

**Authors:** Alicia M Chenoweth, Sandra Esparon, Bruce D Wines, Janine Schuurman, Aran F Labrijn, P Mark Hogarth

**Affiliations:** ^1^ Immune Therapies Group Burnet Institute Melbourne VIC Australia; ^2^ Department of Immunology and Pathology, Central Clinical School Monash University Melbourne VIC Australia; ^3^ Department of Clinical Pathology University of Melbourne Parkville VIC Australia; ^4^ Genmab Utrecht The Netherlands; ^5^ Present address: St. John's Institute of Dermatology, School of Basic & Medical Biosciences Breast Cancer Now Research Unit, School of Cancer & Pharmaceutical Sciences King's College London London UK

**Keywords:** antibody Fc engineering, CD28 super‐agonism, cytokine release syndrome, inhibitory FcR, monoclonal antibody therapeutics

## Abstract

The agonistic action of several immunomodulatory monoclonal antibodies (mAbs) requires both target antigen binding and clustering of this mAb:target complex by the Fcs interacting with Fcγ receptors (FcγRs), in particular FcγRIIb, on neighboring bystander cells. Fc mutations were made in the immunoglobulin G4 (IgG4)‐based TGN1412 anti‐CD28 mAb to define the role of FcγR interactions in its “super‐agonist” activity. The dual mutation, IgG4‐ED^269,270^AA, ablated interaction with all human FcγRs and agonistic action was consequentially lost, confirming the FcγR dependence on the action of TGN1412. The IgG4 lower hinge region (F^234^L^235^G^236^G^237^) was modified by L^235^E mutation (F^234^E^235^G^236^G^237^), a mutation commonly used to ablate FcγR binding, including in approved therapeutic mAbs. However, rather than ablating all FcγR binding, IgG4‐L^235^E conferred specific binding to FcγRIIb, the inhibitory Fc receptor. Furthermore, in combination with the core hinge‐stabilizing mutation (IgG4‐S^228^P, L^235^E), this mutation increased affinity for FcγRIIb compared with wild‐type IgG4. In addition to having FcγRIIb specificity, these engineered TGN1412 antibodies retained their super‐agonistic ability, demonstrating that CD28‐ and FcγRIIb‐specific binding are together sufficient for agonistic function. The FcγRIIb‐specific nature of IgG4‐L^235^E has utility for mAb‐mediated immune agonism therapies that are dependent on FcγRIIb interaction and of anti‐inflammatory mAbs in allergy and autoimmunity that harness FcγRIIb inhibitory signaling.

## Graphical abstract

Contrary to the long‐standing assumption that the L^235^E mutation of the lower hinge of immunoglobulin G4 monoclonal antibodies (mAbs) ablates Fcγ receptor (FcγR) interaction, FcγRIIb binding specificity is conferred. Moreover, use of the L^235^E mutation for Fc effector silencing is problematic as we also show that TGN1412 super‐agonism is retained. This mutation is also present in some approved mAbs. Nonetheless, the FcγRIIb specificity conferred by L^235^E mutation provides a novel basis for development of mAbs designed specifically to exploit FcγRIIb biology.

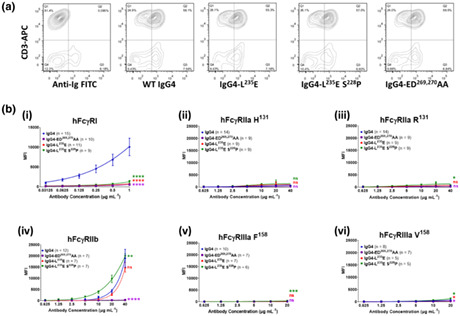

## INTRODUCTION

Monoclonal antibodies (mAbs) are potent and effective biotherapeutics used in a broad range of diseases.^1^ Most therapeutic mAbs are based on human immunoglobulin G1 (IgG1) and can interact with the immune system's effector molecules. However, for some therapeutic approaches, engaging effector molecules, such as Fcγ receptors (FcγRs), is redundant or even detrimental. A common strategy to minimize FcγR interaction and associated effector functions is the use of IgG2 or IgG4 backbones which have naturally restricted FcγR binding specificity.^2^ This strategy has been successful for the development of many therapeutic mAbs, for example, the IgG4‐based anti‐interleukin‐13 mAb tralokinumab^3^ or the IgG2‐based anti‐interleukin‐17Rα mAb brodalumab.^4^ However, IgG4 binds to FcγRIIb and is also a high‐affinity ligand for the activating FcγRI, and so is not an optimal backbone if a truly nonfunctional Fc is desired. Indeed, in its first human trial, the agonistic IgG4‐formatted anti‐CD28 TGN1412 mAb^5^ induced a life‐threatening cytokine storm and multi‐organ failure involving FcγRIIb acting as a scaffold for TGN1412 and crosslinking of CD28 on the T cell surface.^6^

A more direct approach to ablate FcγR interaction is mutation of the Fc domain creating “FcγR‐null” mAbs.^2^, ^7^, ^8^, ^9^ One widely used modification for this purpose is the L^235^E mutation of the lower hinge, which was first used in OKT3, a mouse anti‐human‐CD3 mAb associated with severe cytokine storm.^10^ This mutation eliminated T cell activation presumably by ablation of FcγR binding.^10^ The L^235^E mutation has since been incorporated into clinically approved mAbs, namely, sutimlimab, an anti‐C1s mAb formatted on a hinge‐stabilized IgG4 backbone; durvalumab^11^ and anifrolumab,^12^ which are anti–programmed death‐ligand 1 and anti‐interferon alpha receptor mAbs, respectively; and recently in the severe acute respiratory syndrome coronavirus 2 neutralizing mAb cocktail of tixagevimab/cilgavimab formatted on an IgG1 backbone also containing IgG4‐like modifications of the lower hinge (L^234^F) and F/G loop (P^331^S).^13^, ^14^

In an initial evaluation of the mechanism of the TGN1412‐induced cytokine storm, the TGN1412 IgG4‐L^235^E FcγR‐null control was as potent as TGN1412.^15^ However, inconsistencies are apparent in subsequent evaluations of the mechanistic basis of the TGN1412 cytokine storm which suggested that FcγRIIb interaction was necessary for the severe response.^6^

In this study, we investigated the effect of the L^235^E mutation on the super‐agonist activity and FcγR interactions of the IgG4 anti‐CD28 mAb TGN1412. Notably, we found that both IgG4‐L^235^E and IgG4‐S^228^P, L^235^E were not Fc binding‐inactive but exhibited specific binding to FcγRIIb and thereby retained super‐agonist function.

## RESULTS AND DISCUSSION

**Figure 1:**
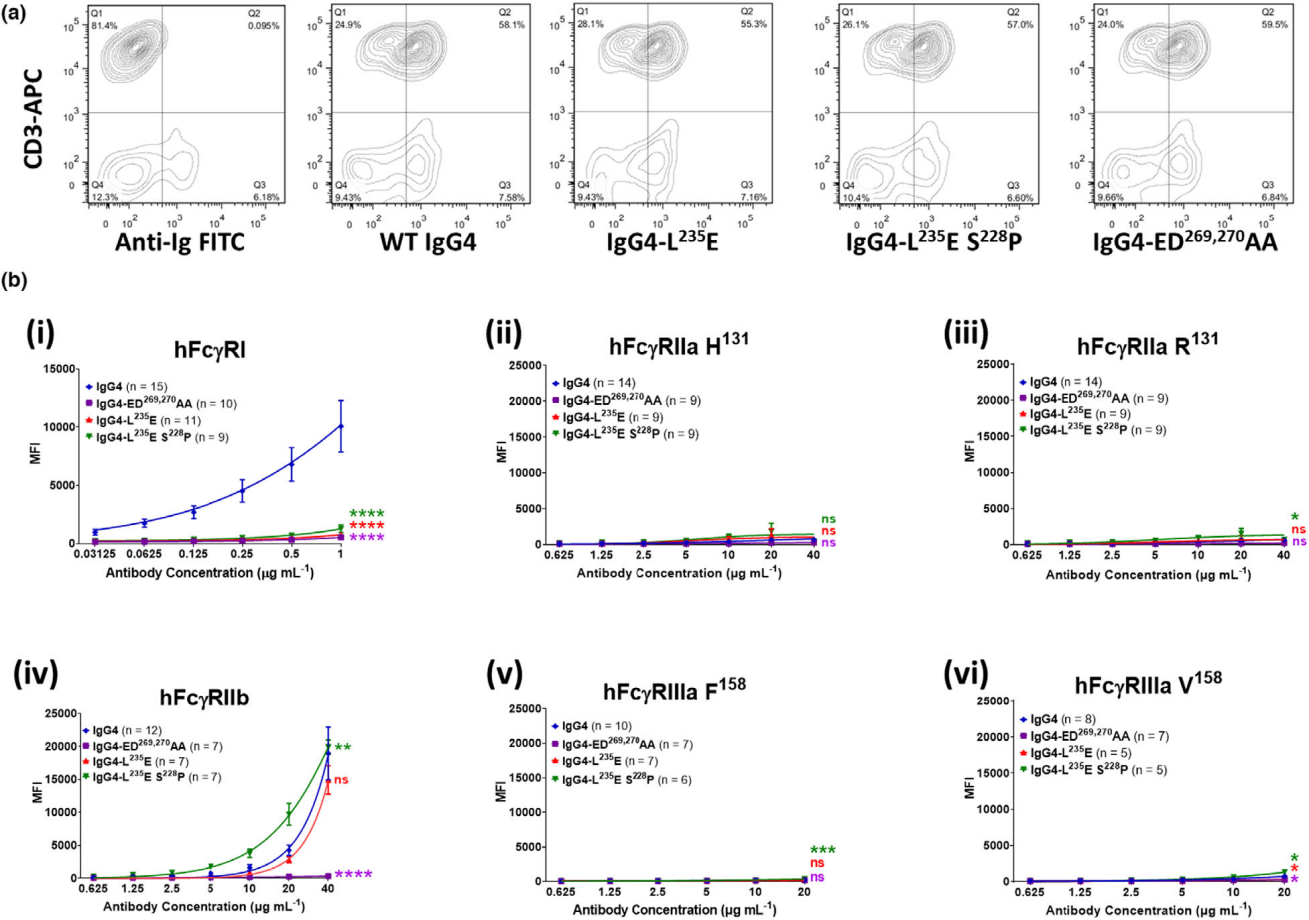
CD28‐ and FcγR‐binding properties of anti‐CD28 wild‐type and mutant antibodies. **(a)** Anti‐CD28 mAb mutants, detected using anti‐human Ig‐FITC (*x* axis), binding to human PBMCs costained with mouse anti‐CD3‐APC, as a marker for T cells (*y* axis). Data are representative of three independent experiments. **(b)** Binding of anti‐CD28 IgG4 mAbs to human FcγRs as detected by flow cytometry. **(i)** FcγRI binding was determined by incubating cells with monomeric IgG mutants (0.03–1 μg mL^−1^) and detected using Alexa Fluor 647–conjugated F(ab′)_2_ fragments of anti‐human IgG F(ab′)_2_. **(ii–vi)**, the binding of complexed CD28 mAbs to low‐affinity receptors FcγRIIa, FcγRIIb and FcγRIIIa was performed by preincubating antibodies (20 μg mL^−1^) with Alexa Fluor 647–conjugated F(ab′)_2_ fragments of anti‐human IgG F(ab′)_2_ (10 μg mL^−1^), then titrating these complexes and incubating with the FcR cells. Analysis was performed with FlowJo. Data are the mean ± standard error of the mean for 5–15 experiments analyzed by two‐way ANOVA with Dunnett's multiple comparisons test, comparing the main column effect with unmodified IgG4; ns, not significant, *P ≤ 0.05, **P ≤ 0.01, ***P ≤ 0.001, ****P ≤ 0.0001. APC, allophycocyanin; FcγR, Fcγ receptor; FITC, fluorescein isothiocyanate; Ig, immunoglobulin; mAb, monoclonal antibody; MFI, mean fluorescent intensity; PBMC, peripheral blood mononuclear cell; WT, wild‐type.

The anti‐CD28 specificity of super‐agonist antibody TGN1412 was formatted as wild‐type (WT) IgG4 or three IgG4 mutants as follows: the presumed “FcγR‐inactive” lower hinge mutant L^235^E (IgG4‐L^235^E); this mutation together with the stabilizing core hinge mutation, S^228^P, (IgG4‐L^235^E, S^228^P) and a novel FcγR‐null mutant IgG4‐ED^269,270^AA. The WT and Fc‐mutated mAbs bound equivalently to CD28 on peripheral T cells at saturating concentrations (Figure 1a).

The binding to the individual cell surface–expressed human FcγRs revealed surprising differences (Figure 1b). As expected, the monomeric WT IgG4 bound to the high‐affinity FcγRI (Figure 1b‐i) and, when formed into an immune complex, bound avidly to FcγRIIb (Figure 1b‐iv), but not to FcγRIIa or FcγRIIIa (Figure 1b‐ii, iii, v, vi). The novel IgG4‐ED^269,270^AA mutation ablated binding to all FcγR (Figure 1b‐i, vi), providing a benchmark for an FcR‐null mAb. Surprisingly, the lower hinge L^235^E mutation did not universally inactivate FcγR binding. Notably, FcγRIIb interaction was retained at levels equivalent to WT IgG4 and was further increased when combined with hinge stabilization (Figure 1b‐iv). As expected, IgG4‐L^235^E binding to FcγRI was ablated, while FcγRIIa and FcγRIIIa binding was negligible (Figure 1b‐i). Thus, the L^235^E mutation confers FcγRIIb specificity.

**Figure 2:**
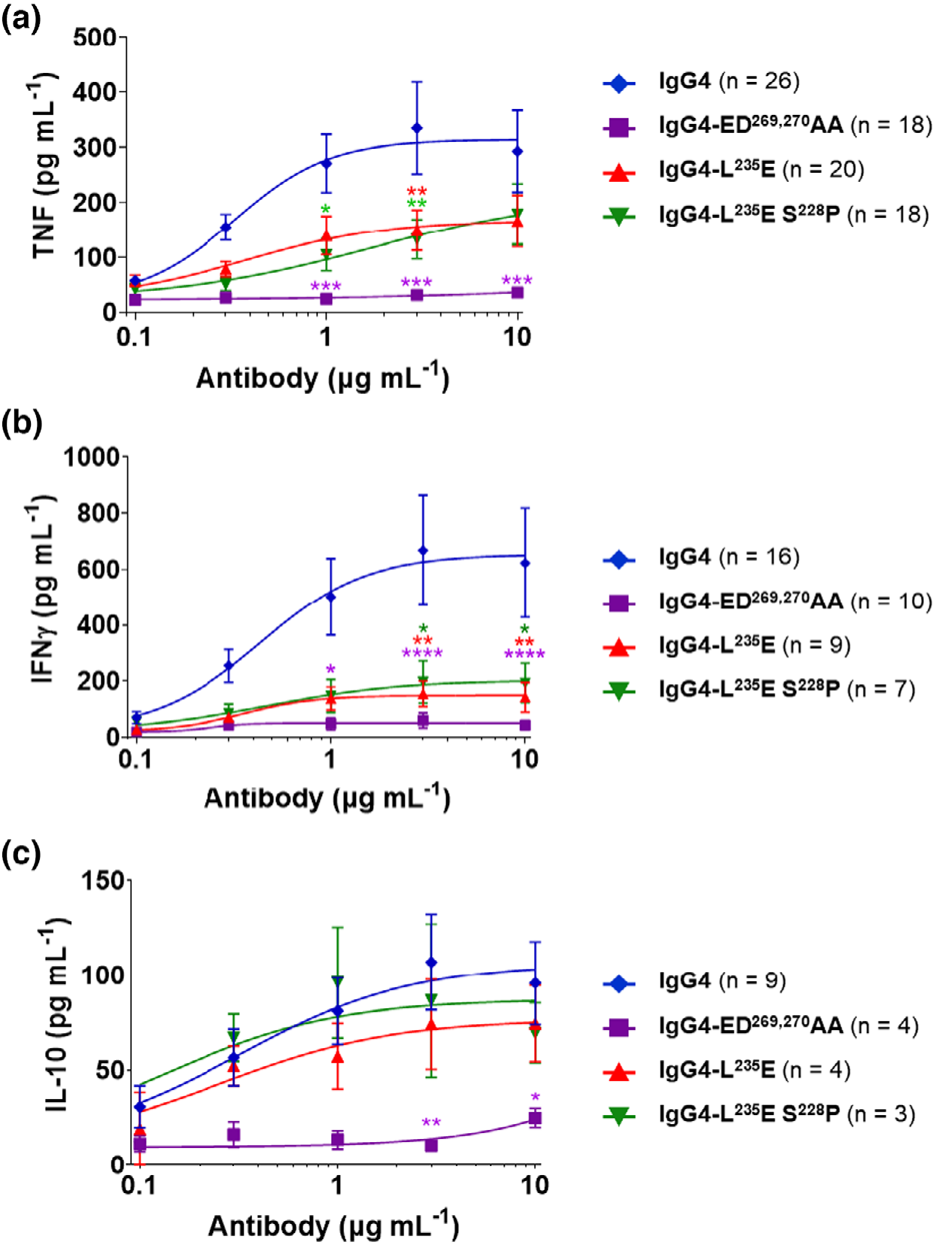
Anti‐CD28 IgG antibody–induced cytokine responses of high‐density cultured PBMCs. PBMCs were cultured at high density (1 × 10^7^ mL^−1^) for 48 h, and then incubated with anti‐CD28 antibodies (0.1–10 μg mL^−1^) for a further 48 h. **(a)** TNF (*n* = 18–26), **(b)** IFNγ (*n* = 7–16) and **(c)** IL‐10 (*n* = 3–9) levels were determined using ELISA. Mean ± standard error of the mean, *n* = 3–26 independent experiments; two‐way ANOVA with Dunnett's multiple comparisons test, comparing to IgG4 WT; *P ≤ 0.05, **P ≤ 0.01, ***P ≤ 0.001, ****P ≤ 0.0001. IFN, interferon; IL, interleukin; TNF, tumor necrosis factor; WT, wild‐type.

The CD28 agonistic activity of the mAbs was evaluated *in vitro*.^16^ The WT IgG4‐ and FcγRIIb‐selective mutants (IgG4‐L^235^E and IgG4‐S^228^P, L^235^E) induced tumor necrosis factor, interferon gamma and interleukin‐10 release, whereas the novel FcγR‐null mutant, IgG4‐ED^269,270^AA, did not (Figure 2). This lack of FcγR binding and lack of agonism by the IgG4‐ED^269,270^AA mutant unequivocally demonstrate the Fc receptor dependence on its agonistic action. This also contrasts with the retention of agonistic potency by the FcγRIIb‐specific L^235^E mutants, which is consistent with the proposed critical role of FcγRIIb in the TGN1412 cytokine storm.^6^ Furthermore, despite similar or increased FcγRIIb‐binding avidity of IgG4‐L^235^E and L^235^E, S^228^P mutants, respectively, over IgG4‐WT, they induced less tumor necrosis factor and interferon gamma compared with WT IgG4 TGN1412, indicating that FcγRIIb‐binding avidity is not the only determinant for maximum agonistic activity. Interestingly, this small reduction in agonism of the IgG4‐L^235^E mutant compared with WT IgG4 TGN1412 contrasts with a previous study that showed near equivalent levels of agonism of WT IgG4 and its IgG4 L^235^E mutant; however, FcγRIIb was not investigated in that study.^15^ The reason for the differences between the two studies is not clear. Altered intrinsic affinity for FcγRIIb is ruled out as TGN1412 and its L^235^E mutant antibodies have equivalent binding avidity to cell surface–expressed FcγRIIb (Figure 1b). More likely are cellular factors that are known to influence TGN1412 agonism, which include the nature of the culture systems such as co‐incubation of peripheral blood mononuclear cell on human umbilical vein endothelial cell monolayers,^15^ which were not used in our study. The levels of cell surface FcγRIIb on the cells under different culture conditions could be another major factor.^6^ Nonetheless, it is clear that L^235^E mutation of the IgG4 anti‐CD28 mAb TGN1412 confers specific binding to the human inhibitory Fc receptor, FcγRIIb, and that its super agonistic action is retained.

We observe FcγRIIb specificity and retention of FcγRIIb‐dependent agonism with FEGG mutation on IgG4 backbone TGN1412. In comparison, the triple mutation L^234^F, L^235^E, P^331^S in IgG1 near‐completely abrogated FcγRI, FcγRIIa, FcγRIIIa and C1q binding,^17^ while the same IgG1 triple mutation of an anti‐tumor necrosis factor mAb did not bind FcγRI, but retained, though reduced, binding to FcγRIIa, FcγRIIb and FcγRIIIa in a high‐avidity formatted binding assay.^18^ Thus, it should be noted that normal human IgG4 already has intrinsically restricted FcR binding specificity interacting only with FcγRIIb and FcγRI. By contrast, human IgG1 is the universal ligand binding to all human FcγR.^2^ It is clear from mutagenesis and structural studies that the interaction between IgG subclasses and the different FcγR is topologically similar; however, the binding of IgG subclasses is nuanced by differences in individual interacting residues in the receptors and IgG ligands. Thus, the effects attributable to the FEGG mutation may be influenced by the choice of IgG backbone and the attributes of FEGG on an IgG4 backbone may not necessarily be recapitulated in IgG1.

Although the L^235^E mutation is historically viewed as an FcγR binding–ablating mutation, it clearly retains specific FcγRIIb binding. This could play a role in the efficacy of some approved therapeutic mAbs using this mutation^11^, ^12^, ^13^ by utilizing the physiologic action(s) of FcγRIIb such as its inhibitory, scaffolding or immune complex removal functions.^19^ Thus, for example, the anti‐severe acute respiratory syndrome coronavirus‐2 mAbs tixagevimab/cilgavimab may harness the potent clearance of mAb–virus immune complexes via the liver sinusoidal endothelium^19^ or interact with FcγRIIb of airway smooth muscle.^20^

Other Fc modifications reported to improve binding to FcγRIIb nonetheless confer very high affinity interactions with some activating‐type FcγRs.^21^ By contrast, FcγRIIb specificity but low affinity of the IgG4‐L^235^E modification makes this format well‐suited to applications where agonistic activity is dependent on avidity conferred by IgG‐Fc complexing, such as scaffolding specifically by FcγRIIb.^22^ Furthermore, there are other potential applications of this IgG4‐L^235^E format in therapeutic mAbs for treating autoimmunity and allergy. Such mAbs could selectively exploit the physiological anti‐inflammatory action of FcγRIIb, such as the suppression of immunoreceptor tyrosine‐based activation motif–dependent activation pathways of the B‐cell receptor or the activating FcγRs, particularly the high‐affinity IgE receptor, FcεRI.^19^, ^23^

## METHODS

### Generation of recombinant mAbs

The TGN1412 VH, VL and kappa CL region sequences^24^ were codon optimized and synthesized together with the IgG4 heavy (H) chain constant domains using gene synthesis services [Thermo Fisher Scientific, Waltham, MA (GeneArt) or Bioneer Corporation, Daejeon, South Korea]. Additionally, several mutants were generated by introducing the L^235^E (IgG4‐L^235^E) mutation, the L^235^E and S^228^P (IgG4‐L^235^E,S^228^P) mutations or the E^269^A and D^270^A (ED^269,270^AA) mutations in the Fc. The H or L chain sequences were subcloned into pCR3 or pcDNA3.4 (Thermo Fisher Scientific) and produced by transient transfection in Expi293 cells and purified by protein A and size exclusion chromatography as described.^25^

### FcγR‐binding assays

FcγR binding was analyzed by flow cytometry.^26^, ^27^ In brief, human FcγRI, FcγRIIa (H^131^ or R^131^ allotype), FcγRIIb and FcγRIIIa (F^158^ or V^158^ allotype) were expressed on FcR‐deficient IIA1.6 cells and binding to the high‐affinity FcγRI or to the low‐affinity FcγRIIa, FcγRIIb and FcγRIIIa was determined using monomeric or complexed anti‐CD28 IgG, respectively, and detected using Alexa Fluor 647–conjugated F(ab′)_2_ fragments of anti‐human IgG F(ab′)_2_(Jackson ImmunoResearch Inc, West Grove, PA).^26^, ^27^

### Cytokine release assays

Peripheral blood mononuclear cells were isolated from healthy human donors or buffy coats obtained from the Australian Red Cross approved by the Alfred Human Research Ethics Committee. All participants gave written informed consent. Peripheral blood mononuclear cells were obtained using Ficoll density gradient centrifugation (Sigma‐Aldrich, St Louis, MI, USA). Any remaining red blood cells were lysed with 155 mm NH_4_Cl, 10 mm KHCO_3_ and 0.1 mm EDTA_2_Na. Prior to CD28–mAb stimulation, peripheral blood mononuclear cells were cultured at high density (1 × 10^7^ cells mL^−1^) in 24‐well plates^16^ for 48 h in Roswell Park Memorial Institute‐1640 supplemented with l‐glutamine, nonessential amino acids, *N*‐2‐hydroxyethylpiperazine‐*N*‐2‐ethane sulfonic acid (HEPES), β‐mercaptoethanol, sodium pyruvate, penicillin/streptomycin and 10% heat‐inactivated AB‐positive human serum. Cells (1 × 10^5^/well) were then transferred to round‐bottomed 96‐well plates with monomeric anti‐CD28 antibodies (0.1–10 μg mL^−1^). After 48 h, supernatants were harvested and cytokine levels were measured by ELISA (ELISAkit.com; Jomar Life Research, Scoresby, Australia).

### Statistics

Binding and cytokine responses were fitted (agonist *versus* response) using GraphPad Prism 9.0 and comparisons with each IgG4 antibody response were by two‐way ANOVA with Dunnett's multiple comparisons test; *P* ≤ 0.05 was considered significant.

## AUTHOR CONTRIBUTIONS

**Alicia Chenoweth:** Conceptualization; data curation; formal analysis; writing – original draft. **Sandra Esparon:** Data curation. **Bruce D Wines:** Conceptualization; data curation; formal analysis; writing – original draft. **Janine Schuurman:** Conceptualization; writing – review and editing. **Aran Labrijn:** Conceptualization; writing – review and editing. **P Mark Hogarth:** Conceptualization; formal analysis; writing – original draft.

## CONFLICT OF INTEREST

The authors declare Alicia Chenoweth, Bruce Wines, Sandra Esparon and P Mark Hogarth are inventors on a PCT application: “Modified immunoglobulin with affinity for FcγRIIb and method of use thereof” owned by the Burnet Institute.

## Data Availability

The data that support the findings of this study are available from the corresponding author upon reasonable request.
